# Unmasking Negative Pressure Pulmonary Edema Following Hanging: A Case Series

**DOI:** 10.7759/cureus.61556

**Published:** 2024-06-02

**Authors:** K K Athish, Guruprasad T J, Spurthy Padmanabha, Harshitha K R

**Affiliations:** 1 Internal Medicine, Sri Devaraj Urs Academy of Higher Education and Research, Kolar, IND; 2 Respiratory Medicine, Sri Devaraj Urs Medical College, Kolar, IND; 3 Pulmonology, Sri Devaraj Urs Academy of Higher Education and Research, Kolar, IND

**Keywords:** acute respiratory distress syndrome (ards), suicide death, suicide mortality, steroids and diuretics, negative-pressure pulmonary edema, hanging, flexible fiberoptic bronchoscopy (ffb), post obstructive pulmonary edema

## Abstract

Pulmonary edema is a rare mechanism of death that develops after partial hanging, a potential complication that physicians should consider early in the management of these patients. This case series discusses the presentation, evaluation, and treatment course of three patients who had attempted suicide by hanging and were admitted to the hospital. These patients were admitted to the intensive care unit after being stabilized and supportive treatment was provided. In all the cases, a radiological scan of the chest revealed diffuse infiltrates consistent with pulmonary edema on both sides, features of which were also noted during a diagnostic bronchoscopy. After providing the best intensive care in the hospital, two patients clinically improved, and one patient succumbed to cardiac arrest. As most patients will be brought dead to the hospital following hanging, negative pressure pulmonary edema remains underdiagnosed. Thus, this case series enumerates the possible etiologies of negative pressure pulmonary edema and its contribution to death following suicidal hanging.

## Introduction

Complete or partial hanging resulting in death is a common encounter in the daily practice of emergency room (ER) consults [1]. Suicidal hanging and death are considered to be a major public concern and are one of the leading causes of death in India, accounting for 57.8% of overall suicides [2]. Patients who survive following injuries sustained by hanging, long enough to reach the hospital are considered as “near hanging” [3]. The injury that results from near hanging, which is a drop from a low height (less than body height), is caused by compression of neurovascular structures in the neck [3]. Among various complications following hanging, an uncommon life-threatening complication is negative-pressure pulmonary edema (NPPE) or post-obstructive pulmonary edema (POPE) initially described in 1977. It is caused by negative intrathoracic pressure resulting from attempted inspiration against an obstructed upper airway [3,4]. NPPE is linked to several causes of upper airway blockage, such as airway infections, tumors, hanging, and laryngospasm during anesthesia and/or after extubation in adults [5]. 

Since most cases of hanging will be brought dead, the incidence of NPPE remains underdiagnosed; therefore, data stating the prevalence of NPPE following hanging remain sparse. Here, we discuss an interesting series of cases of NPPE following near-hanging with details of evaluation, management, and treatment modalities adopted. The probable mechanisms of NPPE in hanging cases have also been reviewed in this article.

## Case presentation

Case 1

**Figure 1:**
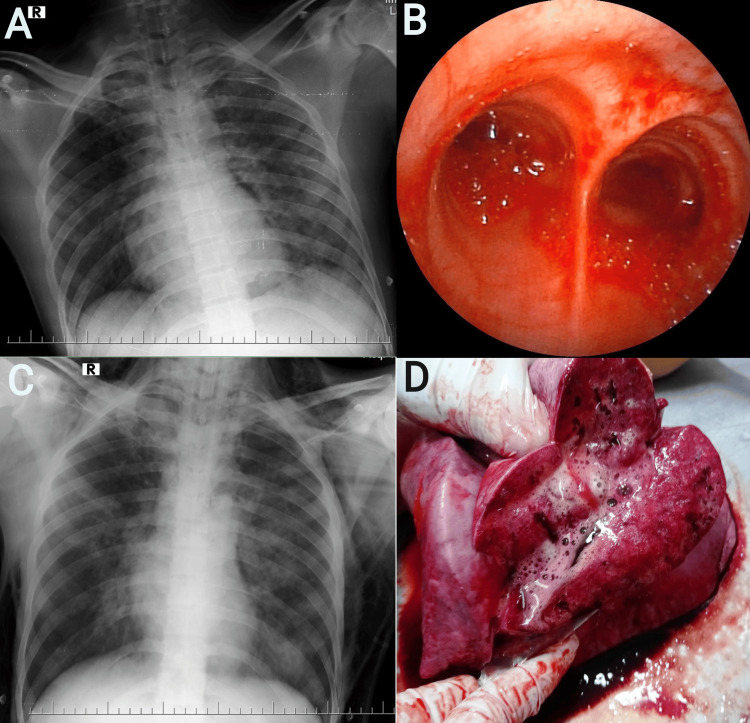
A) Chest radiograph revealed bilateral diffuse infiltrates (left>right) with hilar prominence. B) Flexible bronchoscopy revealed frothy blood flooding until the bilateral mainstem bronchi with airway congestion. C) Chest radiograph revealed bilateral subcutaneous emphysema (left>right) with cephalization of vasculature and persistent pulmonary infiltrates. D) Post-mortem showed frothy secretions from the lungs.

A 23-year-old man weighing 70 kg was brought to the ER with an alleged history of complete hanging from a tree with a rope for an unknown duration of time, and he was rescued by his neighbors. Transportation to the hospital took around 45 minutes. When presented, his Glasgow coma scale (GCS) was 3, with cyanosis with frothing of saliva, and protrusion of the tongue was noted. A complete ligature mark over the neck with subconjunctival hemorrhage was also noted. A cervical collar was placed. On examination, both pupils were 1 mm, sluggishly reacting to light. Doll's eye reflex was sluggish. He had tachypnea, tachycardia, and cold peripheries with blood pressure of 86/44 mmHg and 81% SpO_2_ at room air. Bilateral vesicular breath sounds with coarse crepitations were heard on auscultation. POCUS revealed B-lines suggestive of pulmonary edema and an IVC diameter of 10 mm with preserved left ventricular function. IV fluids were rushed, and the airway was secured by endotracheal (ET) intubation. Copious secretions were present. The electrocardiogram (ECG) revealed sinus tachycardia. The patient was on low tidal volume (Vt) ventilator support as per the acute respiratory distress syndrome network (ARDSNet) protocol. His initial ABG revealed combined metabolic and respiratory acidosis with a lactate of 4.6 PaO_2_/FiO_2_ (P/F ratio) of 126 mmHg and an A-a gradient of 242.3 mmHg. Two-dimensional (2D) echo revealed normal chamber dimensions with trivial MR, mild TR with mild pulmonary artery hypertension (PASP 30 mmHg), and ejection fraction (EF) of 60%. Chest radiograph revealed bilateral diffuse pulmonary infiltrates with hilar prominence (Figure 1A). Head and neck computed tomography (CT) scans were normal. The patient was shifted to the intensive care unit for further treatment. A thorough toxicology workup was negative. Routine laboratory investigations were within normal limits. Regular suctioning revealed frothy blood. On day 2, a flexible fiber optic bronchoscopy (FOB) revealed frothy blood flooding the bilateral mainstem bronchi (Figure 1B), with airway congestion suggesting airway or alveolar hemorrhage in this patient. The patient was initiated on intravenous steroids (methylprednisolone 40 mg twice daily), antifibrinolytics, vitamin K, and continuous infusion of diuretics. Vasopressors were started for hemodynamic support. Bronchoalveolar lavage (BAL) analysis also revealed a high ratio of pulmonary edema fluid to plasma protein with predominant RBCs. Serial ABG analysis revealed worsening hypercapnia, P/F ratio, and lactate levels. On day 3, a check bronchoscopy revealed very minimal secretions marking the resolution of hemorrhage. On day 4, examination revealed decreased breath sounds heard over the left side, and the patient then gradually developed massive subcutaneous emphysema with crepitus extending from the neck to the groin and scrotum. CT thorax revealed left-sided pneumothorax with pneumomediastinum, and subcutaneous emphysema over intermuscular planes of neck spaces and lateral chest wall on the left side with bilateral hilar infiltrates suggestive of pulmonary edema. Clinically, the patient's condition was deteriorating despite adequate management. After clinical and radiological (Figure 1C) evaluation, a diagnosis of NPPE with diffuse alveolar hemorrhage was made. The patient's general condition deteriorated and the patient developed bradycardia on day 5. Despite adequate resuscitative measures, the patient succumbed to the complications. Post-mortem findings revealed frothy secretions from the lungs (Figure 1D).

Case 2

**Figure 2:**
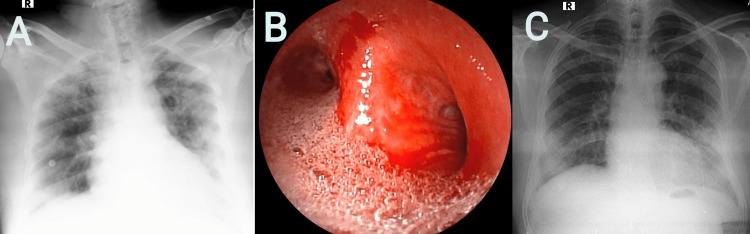
A) Chest radiograph revealed an enlarged cardiac silhouette with bilateral diffuse infiltrates. B) Flexible bronchoscopy revealed frothy secretions flooding the bilateral mainstem bronchi. C) Resolution of pulmonary edema in chest radiograph.

A 45-year-old man was admitted to the ER with an alleged history of hanging for an unknown duration, following rescue by his family member who brought him down from suspension immediately. Transportation to the hospital took about 40  minutes. On arrival, the patient's GCS was 6. Frothing from the mouth, facial congestion and peripheral cyanosis were present. Partial ligature marks around his neck and sub-conjunctival hyperemia were noted. Both the pupils were dilated. The patient was tachypneic with a SpO_2_ of 81%. Moreover, tachycardia with a blood pressure of 84/42 mmHg was recorded. On auscultation of lung fields bilateral extensive crepitations were heard. POCUS revealed B-lines suggestive of pulmonary edema, and IVC diameter of 13 mm with EF of 62%. Resuscitative measures including securing IV access, fluid bolus, and securing the airway by endotracheal intubation were performed, and the neck was stabilized using the cervical collar. The patient was mechanically ventilated and supported with volume-controlled mode. The ECG showed sinus tachycardia without ST abnormalities. ABG revealed mixed metabolic with respiratory acidosis (on 100% FiO_2_, pH 7.07, pO_2_ of 101 mmHg, pCO_2_ 67 mmHg, HCO_3_ 15 mEq/L, and lactate 3.1 mmol/L), A-a gradient was 208.4 mmHg and P/F ratio was 112 mmHg. Upon suctioning of secretions, mild improvement in oxygen saturation was observed. A chest radiograph showed an enlarged cardiac silhouette with bilateral diffuse infiltrates, suggestive of pulmonary edema (Figure 2A). CT study of the brain and cervical spine was unremarkable for traumatic or vascular injury. A high-resolution CT scan of the thorax revealed features suggestive of pulmonary edema. Due to the absence of evidence of other traumatic findings and negative toxicology screens, there was high suspicion for NPPE. All other laboratory parameters were within normal limits. For hemodynamic support, inotropes were initiated. On the next day, FOB revealed frothy secretions flooding the bilateral mainstem bronchi (Figure 2B), following which the patient was initiated on intravenous steroids (methylprednisolone 40 mg twice daily) and diuretics infusion. On day 3, the patient’s respiratory and neurological conditions improved with the resolution of pulmonary edema on the chest radiograph. Resolution of respiratory failure with normalization of pH and lactate was noted in serial ABGs. On day 4, endotracheal secretions were reduced, with the clinical and radiological resolution of pulmonary edema (Figure 2C), following which the patient was extubated.

Case 3

**Figure 3:**
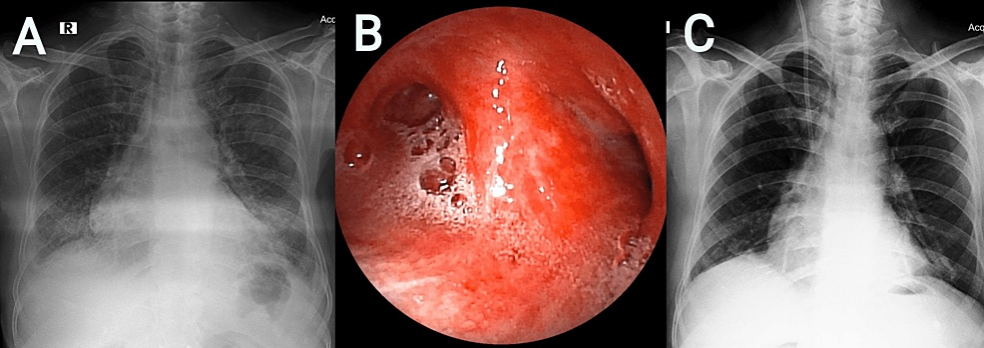
A) Chest radiograph revealed bilateral diffuse infiltrates. B) Flexible bronchoscopy revealed frothy secretions. C) Resolution of pulmonary edema in chest radiograph.

A 52-year-old man landed up in the ER with an alleged history of hanging following a family problem. He was rescued by neighbors who brought him down from suspension. The patient was brought to the hospital in 30 minutes. The duration of the hanging was unknown. During examination in the ER, he had partial ligature marks around his neck, he was restless, poorly responding to oral commands with GCS 7, and frothy secretions from his mouth were noted. Ligature marks were evident over the neck. The patient had tachycardia, tachypnea, and hypotension on examination, and SpO_2_ was 78%. POCUS revealed B-lines suggestive of pulmonary edema, with preserved left ventricular (LV) function (EF 60%) and IVC diameter of 14 mm. Resuscitative measures including securing IV access, fluid bolus, and airway management were performed. Chest imaging was suggestive of pulmonary edema (Figure 3A). The ET tube was secured and the neck was stabilized using a cervical collar. The patient was on low tidal volume (Vt) ventilator support as per the ARDSNet protocol. ECG revealed tachycardia with no ST abnormalities. Regular suctioning was provided because of increased frothy secretions. On 100% FiO2, ABG revealed mixed metabolic and respiratory acidosis with 2.6 mmol/L lactate, the A-a gradient was 220 mmHg and the P/F ratio was 132 mmHg. Ultrasonography of the neck revealed no injuries to the vascular structures. Traumatic or vascular injuries of the cervical spine were ruled out by CT study. However, the HRCT thorax revealed pulmonary edema. Serial ABG analysis showed persistent hypercapnia. FOB revealed frothy secretions in bilateral airways (Figure 3B). After a thorough evaluation, a provisional diagnosis of NPPE was made and the patient was placed on IV corticosteroid (methylprednisolone 40 mg twice daily) and diuretics infusion. On day 3, the patient improved clinically and was extubated. On day 4, radiological resolution was also noted (Figure 3C).

## Discussion

Pathophysiology of morbidity and mortality following hanging as described in the literature are mechanical compression of the neck structures, mainly airway and neurovascular structures resulting in cerebral hypoxia, as well as local injuries, such as fracture of thyroid cartilage/hyoid bone and laryngeal rupture. In addition, hanging can result in laryngeal edema, loss of cervical muscle tone causing delayed airway obstruction, and increased vagal tone from carotid sinus stimulation. Hanging also causes pulmonary complications like ARDS, NPPE, or POPE, which is caused by negative intrathoracic pressure resulting from attempted inspiration against an obstructed upper airway, or generalized vasoconstriction from centrally mediated sympathetic discharge, and secondary cerebral injury from cerebral edema and cerebral hypoxia from arterial dissection or spasm, or subarachnoid hemorrhage [3]. To conclude, death from hanging is usually caused by one of the mentioned mechanisms: (1) direct neurologic damage; (2) mechanical compression of the neck tissues, primarily airway or arteriovenous structures; (3) activation of vasoactive centers in the large vessels leading to cardiac arrest [3].

The overall survival rate of near-hanging ranges between 70% and 100%, although various factors predicting the clinical outcomes have been described, GCS is considered the most contentious of all the criteria. In three case series, independent researchers have shown that a GCS score of 3 at presentation is a reliable indicator of a poor clinical outcome [6-8]. Similarly, a case series reported by Karanth et al. indicated a substantial correlation between a GCS score of less than 7 at presentation and a poor clinical outcome [9]. Contact with the ground during hanging, drop height greater than body height, long duration of hanging, presentation later than 4 hours, hypotension upon arrival [9], the need for cardiopulmonary resuscitation at presentation, circumferential ligature mark indicating severe cerebral anoxia and artery blockage [6], the evidence of cervical spine injuries [8], anoxic brain injury, presence of cerebral edema on CT scan [7], and a PaO2/FiO2 ratio <100 mmHg (severe ARDS) at admission [10] are all predictive factors of poor clinical outcome. Even though the documented survival rates for patients with GCS scores of 3 upon presentation are only up to one-third, regardless of their score it is advised to vigorously resuscitate all such patients [3].

We need to distinguish between the two major types of pulmonary edema, the central-neurogenic origin from the one caused by increased intrathoracic pressure. Two distinct subclasses of NPPE have been described. First, type I is associated with severe inspiratory effort in acute airway obstruction, while type II occurs after the resolution of chronic partial airway obstruction (e.g., adenoidectomy and laryngeal mass resection) [11]. The onset of type I POPE is rapid, but it can occasionally take longer. It is still unclear how exactly pulmonary edema develops following rescue from strangulation or hanging [12]. Postulates suggest brain hypoxia releases vasoactive chemicals, leading to pulmonary vasoconstriction, pulmonary hypertension, and lung congestion. Damage to capillary membranes or lung hyperemia may cause pulmonary edema [12]. Upon deep inspiration against a closed glottis (modified Müller maneuver) or occluded airway, negative inspiratory pressure is generated. The intrathoracic pressure in acute upper airway obstruction is >-100 cm H_2_O, while the normal inspiratory intrathoracic pressure is between -2 and -5 cm H_2_O. The negative inspiratory pressure generated by young people is extremely high with up to a maximum of -140 cmH_2_O. The capillary and alveolar epithelial barriers are disrupted by the intrathoracic transpulmonary negative pressure, which lowers alveolar-capillary membrane pressure leading to blood vessel exudation, allowing red blood cells (RBCs) to seep into the alveoli, causing hemorrhage of lungs or bronchi. Norepinephrine release in response to hypoxia, hypercapnia, and agitation causes a rise in systemic arterial pressure, which in turn causes an increase in pulmonary vascular congestion [5,13].

A large sympathetic discharge mediated by the central nervous system causes neurogenic pulmonary edema. As a result of this discharge, blood is shunted from the high-pressure systemic circulation to the low-pressure pulmonary circulation, causing severe temporary and widespread vasoconstriction. Hypoxic pulmonary vasoconstriction increases capillary permeability. Neurogenic pulmonary edema is frequently seen following a catastrophic brain injury that is typically fatal. Patients with NPPE following strangulation injury probably have a less dire prognosis than those with neurogenic pulmonary edema [11].

As was the case with our patient, patients with NPPE typically have sudden dyspnea with severe hypoxia. Still, the temporal association between hanging and symptom development should be overlooked to consider the diagnosis. All patients with suicidal near hanging need to be aggressively handled including those with significant early neurological impairments and/or respiratory distress irrespective of the outcome.

Treatment of NPPE involves maintaining a patent airway, oxygen supplementation, careful monitoring, and PEEP through endotracheal intubation or noninvasive ventilation as guided by clinical evaluation and ABG, reserving mechanical ventilation to non-responsive cases [14]. Steroids have long been investigated as a possible treatment for both early- and late-phase ARDS due to their ability to reduce the pro-inflammatory cytokine response. Dependent lung regions consolidate during ARDS; this condition is exacerbated by the weight of the heart compressing nearby lung regions. Prone positioning improves ventilation, reduces stress, and alleviates stress by suspending the lung's weight from a larger chest wall. This evenly distributes perfusion, leading to improved V/Q matching and gas exchange. However, proning is contraindicated in the presence of cervical injuries. To prevent mechanical ventilation-induced lung injury "permissive atelectasis," high PEEP levels are applied in conjunction with lower tidal volumes to avoid recurrent alveolar closure and opening [15]. New treatments such as facedown positioning, inhaled nitric oxide, high-frequency breathing, surfactant therapy, and extracorporeal membrane oxygenation are currently being experimented with [16]. Research has been conducted on the use of bronchodilators in pulmonary edema, as studies suggest that bronchodilators, including β-agonists, could promote alveolar fluid clearance to lessen pulmonary edema symptoms [17]. 

While this case series offers a valuable glimpse into the rare complication of NPPE following hanging, it is crucial to acknowledge its inherent limitations. The analysis is based on only three patients, which restricts the generalizability of the findings to the broader and diverse population. Furthermore, the scarcity of data in the literature likely arises from the tragic reality that most hanging victims are deceased upon arrival, leading to a potential underdiagnosis of NPPE. Future prospective studies are needed to solidify our understanding and refine treatment protocols for this serious and potentially fatal complication. Such endeavors could not only strengthen the current evidence but also pave the way for the development of more effective treatment guidelines for NPPE in the context of hanging.

## Conclusions

Although hypoxic injury is the major cause of death in hanging victims, NPPE is a commonly missed complication of hanging caused by obstruction of the upper airway. In a case of near hanging who has respiratory distress, it is emphasized to undergo CT thorax to investigate for pulmonary edema. However, in some cases, inadequate response to mechanical ventilation may necessitate bronchoscopy for further evaluation. Hence, these uncommon complications should be considered in the management of partial hanging. The possibility of NPPE in any case of hanging must be made clear to clinicians. Early management should be initiated when NPPE is suspected in hanging cases, for rapid resolution and to improve the prognosis.
